# Regional pleural strain measurements during mechanical ventilation using ultrasound elastography: A randomized, crossover, proof of concept physiologic study

**DOI:** 10.3389/fmed.2022.935482

**Published:** 2022-09-15

**Authors:** Martin Girard, Marie-Hélène Roy Cardinal, Michaël Chassé, Sébastien Garneau, Yiorgos Alexandros Cavayas, Guy Cloutier, André Y. Denault

**Affiliations:** ^1^Department of Anesthesiology, University of Montreal Hospital, Montréal, QC, Canada; ^2^Division of Critical Care, Department of Medicine, University of Montreal Hospital, Montréal, QC, Canada; ^3^University of Montreal Hospital Research Center, Montréal, QC, Canada; ^4^Laboratory of Biorheology and Medical Ultrasonics, University of Montreal Hospital Research Center, Montréal, QC, Canada; ^5^Department of Medicine, University of Montreal, Montréal, QC, Canada; ^6^Department of Medicine, Sacré-Coeur Hospital of Montréal, Montréal, QC, Canada; ^7^Department of Radiology, Radio-Oncology and Nuclear Medicine, Institute of Biomedical Engineering, University of Montreal, Montréal, QC, Canada; ^8^Department of Anesthesiology, Montreal Heart Institute, Montréal, QC, Canada

**Keywords:** mechanical ventilalion, ventilator-induced lung injury, general anesthesia, lung imaging, pulmonary strain, ultrasound elastography

## Abstract

**Background:**

Mechanical ventilation is a common therapy in operating rooms and intensive care units. When ill-adapted, it can lead to ventilator-induced lung injury (VILI), which is associated with poor outcomes. Excessive regional pulmonary strain is thought to be a major mechanism responsible for VILI. Scarce bedside methods exist to measure regional pulmonary strain. We propose a novel way to measure regional pleural strain using ultrasound elastography. The objective of this study was to assess the feasibility and reliability of pleural strain measurement by ultrasound elastography and to determine if elastography parameters would correlate with varying tidal volumes.

**Methods:**

A single-blind randomized crossover proof of concept study was conducted July to October 2017 at a tertiary care referral center. Ten patients requiring general anesthesia for elective surgery were recruited. After induction, patients received tidal volumes of 6, 8, 10, and 12 mL.kg^–1^ in random order, while pleural ultrasound cineloops were acquired at 4 standardized locations. Ultrasound radiofrequency speckle tracking allowed computing various pleural translation, strain and shear components. We screened 6 elastography parameters (lateral translation, lateral absolute translation, lateral strain, lateral absolute strain, lateral absolute shear and Von Mises Strain) to identify those with the best dose-response with tidal volumes using linear mixed effect models. Goodness-of-fit was assessed by the coefficient of determination. Intraobserver, interobserver and test-retest reliability were calculated using intraclass correlation coefficients.

**Results:**

Analysis was possible in 90.7% of ultrasound cineloops. Lateral absolute shear, lateral absolute strain and Von Mises strain varied significantly with tidal volume and offered the best dose-responses and data modeling fits. Point estimates for intraobserver reliability measures were excellent for all 3 parameters (0.94, 0.94, and 0.93, respectively). Point estimates for interobserver (0.84, 0.83, and 0.77, respectively) and test-retest (0.85, 0.82, and 0.76, respectively) reliability measures were good.

**Conclusion:**

Strain imaging is feasible and reproducible. Future studies will have to investigate the clinical relevance of this novel imaging modality.

**Clinical trial registration:**

www.Clinicaltrials.gov, identifier NCT03092557.

## Introduction

While often a live-saving therapy and a necessity during general anesthesia, mechanical ventilation may promote lung damage when ill-adapted, a phenomenon called ventilator-induced lung injury (VILI) (1). Some patient populations have been shown to be at higher risk of developing VILI: patients suffering from the acute respiratory distress syndrome (2) and patients requiring one-lung ventilation (3).

Assessing mechanical properties of the lung has been suggested as a promising approach to detect conditions leading to VILI (4) with excessive pulmonary strain thought to be a critical factor in its development (5, 6). Unfortunately, pulmonary strain is non-trivial to measure at the bedside (7) and most techniques measure global pulmonary strain, an oversimplification of the complex lung physiology. Areas of higher regional pulmonary strain that cannot be assessed using global measures have been implicated in the development of pulmonary inflammation (8). Detecting these areas of higher regional pulmonary strain may enable identification of patients at higher risk of VILI who have seemingly benign global strain measures and yet may benefit from further strain-lowering interventions (9).

While magnetic resonance imaging (10), positron emission tomography (11) and thoracic computed tomography (9) are currently available in a research setting to measure regional pulmonary strain, these techniques are particularly burdensome, subject patients to the dangers of intra-hospital transport (12), expose patients to ionizing radiations (13) and haven’t been validated against a gold standard nor have they been shown to be associated with clinical outcome. Electrical impedance tomography is an increasingly popular monitoring modality in ventilated patients (14) and has the advantage of being a bedside and radiation-free imaging modality. Unfortunately, electrical impedance tomography cannot currently measure regional pulmonary strain and requires costly dedicated equipment that is currently unavailable in most operating rooms and intensive care units. Consequently, all of the above modalities have major drawbacks that limit their use and validity.

Lung ultrasonography, a bedside, precise, repeatable, easy to learn, and low-cost exam (15), is already used to monitor pulmonary aeration during mechanical ventilation (16). Preliminary works hint that it may be an interesting avenue to measure regional pleural strain as a surrogate for regional pulmonary strain (17, 18). Ultrasound (US) systems, now standard equipment in most operating rooms and intensive care, could make bedside regional pleural strain measurement commonplace. However, prospective feasibility data is lacking.

The objective of this study was to assess the feasibility and reliability (intraobserver, interobserver, and test-retest) of pleural strain measurements by ultrasound elastography. Moreover, we sought to determine if elastography indices of pleural translation, strain and shear would correlate with varying tidal volumes.

## Materials and methods

This report was redacted following the CONSORT 2010 extension to randomized crossover trials statement (19).

### Study design

This study was a pilot single-center, single-blind, randomized, four period crossover trial. A crossover design was chosen for this study to improve its power while dropouts and a potential carryover effect were not expected. The protocol was approved by the ethics committee of the Centre hospitalier de l’Université de Montréal (16.386) and registered at ClinicalTrials.gov (NCT03092557, registered March 28th, 2017). Written informed consent was obtained from all study participants.

### Study population

Between July and October 2017, adult patients with healthy lungs who were scheduled to undergo an elective surgery under general anesthesia requiring endotracheal intubation and muscle relaxation were screened for inclusion. Patients were recruited at the Centre Hospitalier de l’Université de Montréal (Montréal, Canada), a tertiary care referral center. Patients with healthy lungs were defined as: no active or past history of smoking, no previous intrathoracic procedure, no known pulmonary disease, no oxygen requirement and metabolic equivalent task (METS) greater than or equal to 4. To provide optimal imaging conditions for this pilot study, obese patients (body mass index > 30 kg.m^–2^) were excluded.

### Interventions

All patients were pre-oxygenated in the supine position with 100% oxygen for 3 min without any continuous positive airway pressure. While specific doses were not protocolized, general anesthesia induction was performed using standard doses of propofol and fentanyl. Rocuronium was used in all cases to facilitate tracheal intubation. Anesthesia was maintained with desflurane or sevoflurane. All patients were ventilated with Datex-Ohmeda Aestiva 3000 machines (GE Healthcare, WI, United States) using volume-controlled ventilation, an inspired oxygen fraction (F_*i*_O_2_) of 40–50%, a respiratory rate of 12 min^–1^, an inspiratory to expiratory ratio of 1:2, no inspiratory pause and a positive end-expiratory pressure of 6 cm H_2_O.

After anesthesia induction, patients were administered tidal volumes of 6, 8, 10, and 12 mL.kg^–1^ predicted body weight (20) in random order (Supplementary Figure 1). For each tidal volume, mean expired tidal volume of 3 consecutive breaths were collected and the pleura was imaged at 4 predetermined anatomical locations: Left and right 3rd intercostal space at the mid-clavicular line, and left and right 8th intercostal space at the posterior axillary line. The correct intercostal spaces were identified by sliding the ultrasound transducer from the clavicle downwards and visually counting rib spaces. For each tidal volume and anatomical location tested, 3 ultrasound radiofrequency cineloops at a frame rate of 30 Hz were acquired over 3 separate respiratory cycles. All cineloops were saved to digital format for offline analysis. Interobserver reliability was assessed by repeating cineloop acquisition by a second blinded observer (SG) for the 10 mL.kg^–1^ predicted body weight tidal volume. Immediately after, test-retest reliability was also assessed by repeating cineloop acquisition by the first observer (MG) for the 10 mL.kg^–1^ predicted body weight tidal volume.

### Lung ultrasonography

Lung ultrasonography was performed by experienced lung echographists (MG and SG for repeated measures with 8 and 1 years of experience) using a Terason T3000cv scanner (Teratech Corporation, Burlington, MA) and a 12 MHz transducer (probe #12L5). Initial depth of field was 4 cm and adjusted as needed to position the pleura between half to three-quarters of the screen. A single focal zone was placed nearest to the pleura. With the marker pointing toward the head, the probe was oriented perpendicular to the ribs with the pleura as horizontal as possible.

### Elastography

B-mode images were reconstructed from radiofrequency data (Supplementary Figure 2A). For each cineloop, the pleura was segmented manually on a single frame (Supplementary Figure 2B). With the segmented pleura forming the upper boundary, a region of interest (ROI) of a fixed depth of 2 mm was defined (Supplementary Figure 2C). The geometry of the ROI was automatically adapted and tracked throughout the respiratory cycle (21) or, if inadequate, simply copied over from frame to frame. Tracking was considered adequate when the pleural line remained within the ROI at all-time as assessed visually by an experienced lung echographist (MG). Elastography parameters were computed on all cineloops with a visible pleura and an adequately tracked ROI. We used the Lagrangian speckle model estimator to compute tissue translation, strain and shear values (Supplementary Figure 3) (22). We defined translations as rigid displacements produced by lung sliding, strain as expansion or contraction of the pleura from tidal volume administration and shear as the angular deformation.

**FIGURE 1:**
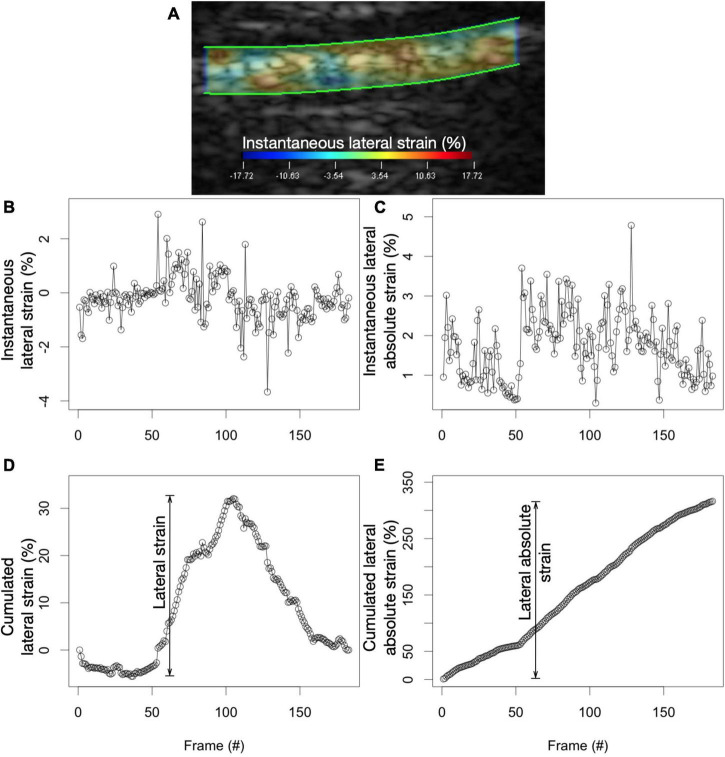
Calculating lateral strain and lateral absolute strain values. **(A)** Instantaneous strain values are computed in all sub-ROIs between consecutive frames of a cineloop. **(B)** By averaging all instantaneous sub-ROI strain values in a single frame, instantaneous strain values for the whole ROI are plotted for all frames of the cineloop. **(C)** The summation of instantaneous strain values produces the cumulative strain of the pleura. Lateral strain is the range of the cumulative lateral strain experienced by the lung in the ROI. **(D)** On the other hand, by averaging all *absolute* sub-ROI instantaneous strain values in a single frame, instantaneous *absolute* strain values for the whole ROI are plotted for all frames of the cineloop. **(E)** The summation of the instantaneous *absolute* strain values produces the cumulative *absolute* strain of the pleura. Lateral *absolute* strain is the range of the cumulative lateral *absolute* strain experienced by the lung in the ROI.

Elastography images and mechanical parameters were computed within the ROI over consecutive frames. We used an implementation of the estimator integrated into a commercial imaging platform (Visual, Object Research Systems, Montréal, Canada) (23). This implementation computes the various elastography parameters using the raw radiofrequency data and not on the reconstructed B-mode images. As such, computed elastography parameters are not affected by gain or image post-processing techniques. Axial and lateral elastography components were determined (“axial” indicating the direction along the US beam and “lateral” indicating the direction perpendicular to it). Considering the planar nature of the pleura and its perpendicular orientation with respect to the US beam, we restricted our analysis to lateral translation, strain and shear components along with the Von Mises strain, a combination of bidimensional strain and shear components. Six elastography parameters were computed per cineloop of a given patient (Figure 1 and Table 1).

**TABLE 1:** Description of elastography parameters.

Parameters (units)	Description
**Lateral**	
**Shift**	
Lateral translation (mm)	Range of the cumulated lateral shift. It represents the range of the distance traveled by the pleura on both sides of its starting point because of lung sliding.
Lateral absolute translation (mm)	Range of the absolute cumulated lateral shift. The absolute cumulated lateral shift was calculated by summation of a per-frame average of the absolute values of all individual sub-ROI computed instantaneous lateral shifts. It is always positive and represents the total distance traveled by the pleura throughout the respiratory cycle because of lung sliding.
**Strain**	
Lateral strain (%)	Range of the cumulated lateral strain. It represents the range of the expansion (or contraction) of the pleura from tidal volume insufflation and exsufflation.
Lateral absolute strain (%)	Range of the absolute cumulated lateral strain. The absolute cumulated lateral strain was calculated by summation of a per-frame average of the absolute values of all individual sub-ROI computed instantaneous lateral strain. It is always positive and represents the total lateral strain (expansion and contraction) experienced by the pleura throughout the respiratory cycle from tidal volume insufflation and exsufflation.
**Shear**	
Lateral absolute shear (%)	Range of the absolute cumulated lateral shear. The absolute cumulated lateral shear was calculated by summation of a per-frame average of the absolute values of all individual sub-ROI computed instantaneous lateral shear. It is always positive and represents the total angular strain (left-sided and right-sided) experienced by the pleura throughout the respiratory cycle from tidal volume insufflation and exsufflation.
**Bidimensional**	
Von Mises strain (%)	Range of the cumulated Von Mises strain. Von Mises strain is a combination of axial and lateral strain and shear components. It is always positive and represents the magnitude of the total strain experienced by the pleura throughout the respiratory cycle from tidal volume insufflation and exsufflation.

### Randomization and blinding

Simple randomization was performed by a statistician not otherwise implicated in this study using a computer random number generator [R v3.4.0, R Core Team, (24); blockrand package v1.3, Snow, (25)]. Concealment was ensured by the use of sequentially numbered sealed and opaque envelopes. Both observers were not blinded to tidal volume during lung cineloop acquisition and during image analysis.

### Objectives

The primary objective was the feasibility of pleural strain measurement using ultrasound elastography. Secondary objectives were: (1) Verifying dose-response with varying tidal volumes and model fit for elastography parameters; (2) measuring reliability (intraobserver, interobserver and test-retest) of elastography parameters.

### Outcomes

The primary outcome was feasibility defined as the percentage of all cineloops mandated by the protocol on which we computed elastography parameters. Secondary outcomes were: (1) Estimated elastography parameter slopes for tidal volume and marginal and conditional coefficients of determination (*R*^2^); (2) intraclass correlation coefficients (ICC) for intraobserver, interobserver and test-retest reliability values of elastography parameters.

### Statistical analysis

We enrolled a convenience sample of 10 patients. For the primary outcome, with 72 potential cineloops per patient, our margin of error is 2.2% for a 95% confidence level and an expected 90% feasibility.

For the first secondary outcomes, computation results of all 6 elastography parameters were modeled. All continuous dependent and independent variables were centered and reduced. This allowed direct comparison of slope estimates from the various models. To simplify models, all repeated measurements performed at a tidal volume of 10 mL.kg^–1^ used to measure interobserver and test-retest reliability values were not included in the models. Linear mixed-effect models were used with elastography parameters as dependent variables and tidal volume, side of measurement (left/right) and gravity dependence of measurement (dependent/non-dependent) as independent variables. Because of the repeated (and thus correlated) nature of measurements per patient and per anatomical location, a random intercept per patient was included in the model as well as a one per anatomical location as a nested grouping factor, whereas triplicate measurements used to measure intraobserver reliability were averaged. Interaction between tidal volume and gravity dependence was included in the model (9). Model assumptions were verified. All six slope estimates were tested for significance. Using Bonferroni’s adjustment, a *p*-value of 0.008 (0.05/6) was considered significant. All other analyses are considered exploratory. To identify elastography parameters with the best dose-response with tidal volumes, absolute values of estimated slopes for significant parameters were ordered. Parameters with highest absolute values of estimated slopes with non-overlapping 95% confidence intervals were selected. Goodness of fit was assessed by the marginal and conditional *R*^2^ (26). To ensure robustness of results, a sensitivity analysis was performed by using the same linear mixed-effect models described above but using a smaller subset of cineloops with better imaging quality. Cineloops with better imaging quality were defined as having a thin and clearly defined pleural line and perfect ROI tracking.

For the second secondary outcome, intraobserver, interobserver and test-retest reliability measures were calculated using ICC type (2,1) (27). As outlined above, cineloops were acquired by different observers while the analysis process was performed by the same one. Bootstrap was performed as a second sensitivity analysis to calculate 95% confidence intervals using 10,000 iterations and the bias-corrected and accelerated method (28). As suggested, ICC values less than 0.5 indicate poor reliability, values between 0.5 and 0.75 indicate moderate reliability, values between 0.75 and 0.9 indicate good reliability and values greater than 0.9 indicate excellent reliability (27). Bland-Altman plots were also performed for intraobserver, interobserver and test-retest reliability. We computed mean bias and 95% limits of agreements for interobserver and test-retest reliability measures (29) but not for intraobserver reliability measure as we could not identify a generally accepted methodology that takes into account triplicate measurements for a single observer.

Results are expressed as mean ± standard deviation or median and interquartile range (25–75%) as appropriate. No imputation for missing values was performed. Statistical analyses were performed using R [v3.4.0, R Core Team, (24)].

## Results

Twelve patients were assessed for eligibility. Two patients were excluded post-enrollment (Supplementary Figure 4): one change in anesthetic plan and one consent withdrawal. Patients’ baseline characteristics are summarized in Table 2.

**TABLE 2:** Patient characteristics.

Variables	Value (*n* = 10)
Age (y)	53 (37–66)
Sex, M/F (no)	5/5
ASA classification, 1/2/3 (no)	3/5/2
Height (cm)	167 (163–177)
Weight (kg)	74 (64–89)
Body mass index (kg.m^–2^)	27 (24–30)
Predicted body weight (kg)	61 (55–72)

All data presented as median (interquartile range) unless otherwise specified.

With our protocol specifying that 72 cineloops would be acquired for each of the 10 patients randomized, 720 elastograms were planned to be computed upon completion of cineloop acquisition. Sixty-two cineloops (8.6%) weren’t subjected to segmentation and tracking: 4 cineloops were not acquired because of a manipulation error, 7 cineloops were incomplete because of a technical issue with the echograph, and 51 cineloops didn’t show sufficient pleura throughout the respiratory cycle. This last problem was only encountered for cineloops acquired over the left 3rd intercostal space at the mid-clavicular line where the heart was mostly seen. Lastly, elastograms were not computed for 6 cineloops because of inadequate tracking of the ROI throughout the respiratory cycle. Elastogram computation was thus performed on 652 (90.6%) cineloops.

**FIGURE 2:**
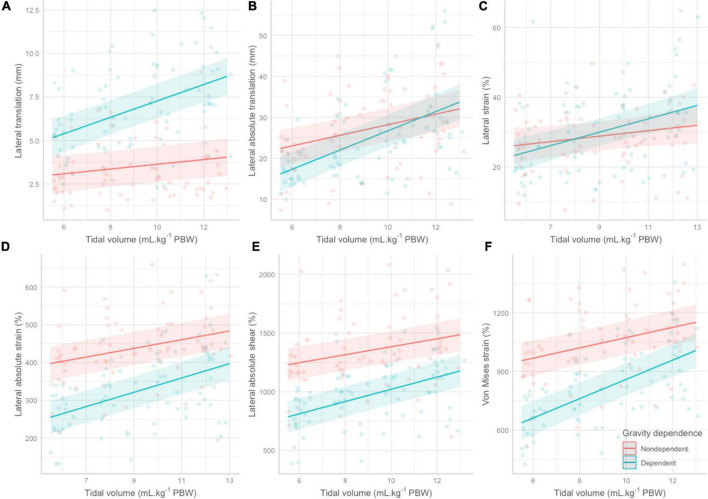
Regression lines, 95% confidence bands and individual data points for all elastography parameters across the various tidal volumes stratified by gravity dependence. **(A)** Lateral translation. **(B)** Lateral absolute translation. **(C)** Lateral strain. **(D)** Lateral absolute strain. **(E)** Lateral absolute shear. **(F)** Von Mises strain. PBW, predicted body weight.

**FIGURE 3:**
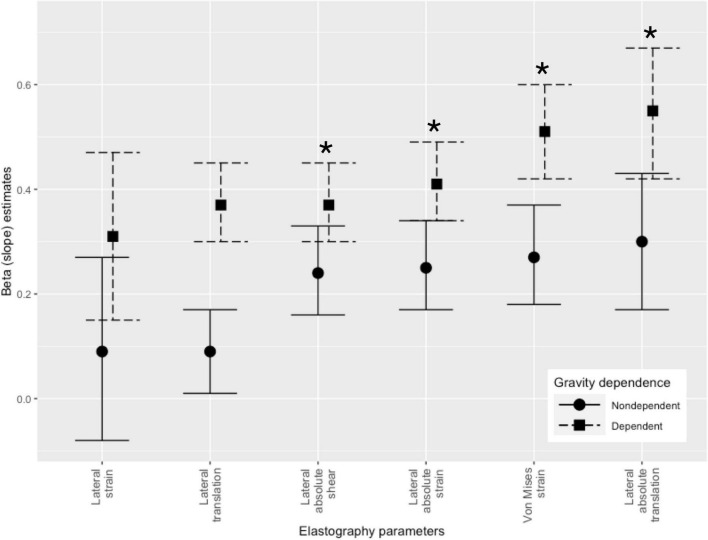
Slope estimates for elastography parameters in increasing order stratified by gravity dependence. Significant parameters are identified by an asterix.

Estimates for fixed effects of all 6 models can be found in Table 3. We observed a significant linear increase in 4 elastography parameters with increasing tidal volume: lateral absolute shear, lateral absolute strain, Von Mises strain and lateral absolute translation (Figure 2). With overlapping 95% confidence intervals, no significant elastography parameter was superior to the other in its ability to capture an increase in tidal volume (Figure 3). Amongst them, lateral absolute shear, lateral absolute strain and Von Mises strain were more precisely predicted by tidal volume compared to lateral absolute translation, as shown by higher goodness of fit (Table 3). The first sensitivity analysis excluding suboptimal images yielded similar results (Supplementary Table 1 and Supplementary Figure 5). When side and gravity dependence of measurements were considered, only gravity dependence seemed to be associated with our results (Table 3). Dependent lung zones had initially lower values for all significant strain elastography parameters, but pleural strain measurements in dependent lung zones increased more rapidly with increasing tidal volume although this was only significantly with the Von Mises strain parameter (Table 3 and Figures 2, 3).

**TABLE 3:** Modeled elastography parameters.

Elastography parameters	b_1_ (slope) estimates for tidal volume		*P*-value	Marginal *R*^2^	Conditional *R*^2^	Left effect estimates	Dependent effect estimates
						
	Non-dependent	Dependent	Slope			(vs. Right)	(vs. Non-dependent)
Lateral strain	0.09 (–0.08 to 0.27)	0.31 (0.15–0.47)	0.3	0.20	0.48	–0.43 (–0.78 to –0.08)	0.64 (0.29–0.99)
Lateral translation	0.09 (0.01–0.17)	0.37 (0.3–0.45)	0.03	0.46	0.89	–0.24 (–0.63 to –0.15)	1.25 (0.86–1.64)
Lateral absolute shear	0.24 (0.16–0.33)	0.37 (0.3–0.45)	<0.0001	0.39	0.89	0.04 (–0.37 to 0.44)	–1.13 (–1.54 to –0.73)
Lateral absolute strain	0.25 (0.17–0.34)	0.41 (0.34–0.49)	<0.0001	0.37	0.88	0.07 (–0.36 to 0.49)	–1.05 (–1.48 to –0.63)
Von Mises strain	0.27 (0.18–0.37)	0.51 (0.42–0.6)	<0.0001	0.40	0.86	0.07 (–0.35 to 0.49)	–1.01 (–1.44 to –0.59)
Lateral absolute translation	0.3 (0.17–0.43)	0.55 (0.42–0.67)	<0.0001	0.21	0.72	0.26 (–0.2 to 0.72)	–0.23 (–0.69 to 0.23)

Ordered by increasing b1 (slope) estimate.

**FIGURE 4:**
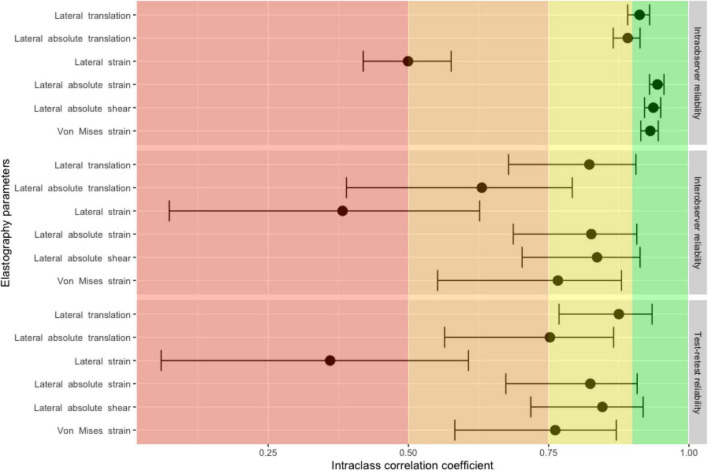
Intraclass correlation coefficients and 95% confidence intervals for intraobserver, interobserver and test-retest reliability measures for elastography parameters. Intraclass correlation coefficients in the red panel indicate poor reliability, values in the orange panel indicate moderate reliability, values in the yellow panel indicate good reliability and values in the green panel indicate excellent reliability.

Measured intraobserver reliability was excellent for lateral absolute shear, lateral absolute strain and Von Mises strain. Interobserver and test-retest measured reliabilities were found to be moderate to good from Von Mises strain and moderate to excellent for lateral absolute shear and lateral absolute strain (Figure 4 and Supplementary Table 2). In our sensitivity analysis, bootstrapping yielded identical estimates (Supplementary Table 3). Bland-Altman plots, mean bias and 95% limits of agreements can be found in Supplementary Table 4 and in Supplementary Figure 6).

## Discussion

We showed preliminary evidence that computing regional pleural translation, strain and shear components is feasible in over 90% of cineloops. Lateral absolute shear, lateral absolute strain and Von Mises strain were more informative in regards to administered tidal volumes than lateral translation, lateral absolute translation and lateral strain. Measured intraobserver reliability was excellent for all significant parameters while interobserver and test-retest measured reliability were found to be moderate to excellent. This supports our hypothesis that lung ultrasound could be used to measure regional pleural strain in mechanically ventilated patients.

While others have previously described ultrasonographically-measured regional pleural strain in humans (17, 18), this study significantly extends previous work by quantifying the impact of tidal volume on regional pleural strain. Despite the absence of a gold standard precluding any firm conclusion about the utility of each elastography parameter, we described an expected dose-response relationship between various strain measurements and administered tidal volumes.

At first glance, it is somewhat surprising that a 2 mm thick ROI delineated from an image generated by a 12 MHz probe could capture physiological signal from the pleura, a structure only 30–40 microns thick (30). With the topmost part of the ROI aligned with the pleura, the image contained within the ROI is actually mainly composed of reverberation artifacts originating from the pleura and other soft tissue/ultrasound beam interfaces (31). These artifacts are most easily seen when contrasting normal lung sliding in M-mode and their disappearance when a pneumothorax occurs (Supplementary Figure 7). We hypothesize that reverberation artifacts in the ROI convey anatomical information on the pleura thus allowing our algorithm to compute elastography parameters. Another possibility would be that imperfect ROI tracking leads to intercoastal muscle being included in the strain calculation. While it is technically possible to measure intercostal muscle strain (32), we do not believe this is the case with our results as patients were paralyzed for induction of general anesthesia.

One important finding is that lateral strain had a poor dose-response with tidal volume despite a time plot of lateral strain that seemed to track delivered tidal volume in many patients (Figure 1C). Perhaps explaining the poor performance of lateral strain compared to lateral absolute strain, we observed small areas of negative strain during lung inflation (Figure 1A). Supporting this hypothesis, zones of local deflation during inspiration have been described in animals (Supplementary Figure 8) (33, 34). Analogous to how lateral strain and lateral absolute strain are calculated, global pulmonary compliance was correlated with the sum of positive values of local compliance, measured by computed tomography, but not with the sum of positive and negative values of local compliance (34). While the exact causes for this phenomenon are still debated, tidal recruitment with gas redistribution may contribute. Discrepancies between lateral strain and lateral absolute strain could signal tidal recruitment and ongoing atelectrauma.

Our study has some important strengths and limitations. First, our results are physiologically plausible with lateral absolute shear, lateral absolute strain and Von Mises strain increasing with increasing tidal volumes. Second, our two sensitivity analyses demonstrate the robustness of results. Third, by performing a randomized crossover study, we ruled out the possibility of an order effect. Fourth, we demonstrated the good to excellent reliability of these measurements. Given the proof-of-concept nature of this study, several limitations should be considered. First, the limited sample size restricts our ability to obtain precise estimates of pleural strain in human. Second, no gold standard for pulmonary strain was available for comparison. While helium dilution or nitrogen-washout techniques could have been used to measure end-expiratory lung volume at each step of our protocol, these methods cannot account for tidal recruitment or overdistension (35). Third, whether regional pleural strain is a good surrogate for regional pulmonary strain remains to be determined. Alveolar deflation kinetics in a small animal model suggest that differences between the lung’s periphery and core regions exist and that these may be altered by disease states (36, 37). On the other hand, lung lesions in another VILI animal model developed predominantly in the subpleural regions (38). Further work will need to be done to determine the clinical usefulness of lung US-measured regional pleural strain. Fourth, flow, pressure and volume curves were not recorded simultaneously with each image acquisition. As such, the precise beginning and end of each respiratory cycle wasn’t exactly known which may have affected our results. The exact tidal volume administered while acquiring cineloops may also have been slightly different from the tidal volume recorded beforehand. Fifth, physiological lung sliding and rib shadows preclude measuring the same pleural surface throughout the respiratory cycle. While we computed strain between two consecutive frames of a given cineloop, the pleura imaged in the ROI at end-inspiration was likely different from the pleura in the ROI at end-expiration. As such, strain measures likely represent average strain values from a pulmonary neighborhood. Sixth, the elastography parameters calculated from the Lagrangian speckle model estimator reflect dynamic pleural strain (pleural deformation from regional tidal volume/gas volume at set positive end-expiratory pressure) and not total pleural strain (pleural deformation from total gas volume/gas volume at functional residual capacity). While experimental evidence suggests dynamic strain is the most important strain component leading to the development of VILI (39–41), the clinical usefulness of lung US-measured regional pleural strain will need to be verified in future studies. Seventh, patients included in this proof-of-concept study were highly selected as we excluded patients with any lung pathology and obese patients. While our first sensitivity analysis that excluded suboptimal images did not show different results (Supplementary Table 1 and Supplementary Figure 5), our results’ external validity remains to be demonstrated and future studies will have to include obese patients and patients with various lung pathologies. Finally, like all ultrasonographic exams, lung ultrasonography is operator dependent. Fortunately, because of lung ultrasonography’s shallow learning curve, it is possible to train a clinician rapidly. As few as 10 supervised exams have been found to be necessary when assessing simple pathologies (42) although interpreting more complicated quantitative aspects of lung US requires a 2-month period and 25 supervised exams (43).

In conclusion, measuring regional pleural strain by ultrasound elastography is feasible. A significant dose-response relationship between tidal volume and lateral absolute shear, lateral absolute strain and Von Mises strain was observed, supporting the hypothesis that regional pleural strain can be measured at the bedside with non-invasive lung ultrasound. Further work will be required to compare elastography parameters to gold-standard strain measurements and how they can be used to tailor mechanical ventilation in the operating room and the intensive care unit to improve patient outcomes.

## Data availability statement

Due to national regulations in the Province of Quebec (Canada), health medical data cannot be made available publicly. However, access to the research dataset is possible for research purpose after appropriate privacy agreements between research parties have been completed. Data access requests may be sent to the corresponding author MG, martin.girard@umontreal.ca, or directly to the CHUM REB (ethique.recherche.chum@ssss.gouv.qc.ca). The R code will be available upon request to the corresponding author.

## Ethics statement

The studies involving human participants were reviewed and approved by the Comité d’éthique de la recherche du CHUM. The patients/participants provided their written informed consent to participate in this study.

## Author contributions

MG, YC, GC, and AD designed the study. MG and SG collected the data. MG, M-HRC, MC, GC, and AD analyzed the data. MG wrote the manuscript. All authors have read and approved the final manuscript.
