# Supplementary material for: Regional pleural strain measurements during mechanical ventilation using ultrasound elastography: A randomized, crossover, proof of concept physiologic study
Source: Front Med (Lausanne). 2022 Sep 15;9:935482. doi: 10.3389/fmed.2022.935482 (PMC9520064; doi:10.3389/fmed.2022.935482)

Supplementary Material

**Table S1. Modeled elastography parameters using reduced dataset**

| Elastography parameters | b_1_ (slope) estimates | | P value | Marginal  R^2^ | Conditional  R^2^ | Left effect estimates | Dependent effect estimates |
| --- | --- | --- | --- | --- | --- | --- | --- |
|  | Nondependent | Dependent | slope |  |  | (vs right) | (vs nondependent) |
| Lateral strain | 0.14  [-0.07 to 0.35] | 0.38  [0.21 to 0.55] | 0.2 | 0.20 | 0.48 | -0.33  [-0.72 to 0.05] | 0.63  [0.24 to 1.02] |
| Lateral translation | 0.11  [0.01 to 0.21] | 0.4  [0.32 to 0.48] | 0.03 | 0.42 | 0.89 | -0.17  [-0.63 to 0.3] | 1.2  [0.73 to 1.67] |
| Lateral absolute shear | 0.33  [0.23 to 0.42] | 0.39  [0.32 to 0.47] | < 0.0001 | 0.43 | 0.90 | 0.1  [-0.36 to 0.57] | -1.16  [-1.63 to -0.69] |
| Lateral absolute strain | 0.33  [0.24 to 0.42] | 0.43  [0.35 to 0.5] | < 0.0001 | 0.40 | 0.91 | 0.15  [-0.32 to 0.62] | -1.07  [-1.54 to -0.6] |
| Von Mises strain | 0.33  [0.23 to 0.43] | 0.51  [0.43 to 0.59] | < 0.0001 | 0.44 | 0.89 | 0.15  [-0.33 to 0.63] | -1.06  [-1.53 to -0.58] |
| Lateral absolute translation | 0.32  [0.18 to 0.46] | 0.52  [0.4 to 0.63] | < 0.0001 | 0.23 | 0.77 | 0.38  [-0.15 to 0.9] | -0.34  [-0.86 to 0.19] |

Parameters ordered as in Table 1 from the main manuscript for easier comparison.

**Table S2. Intraobserver, interobserver and test-retest measured reliability for elastography parameters**

| Elastography parameters | Intraobserver  reliability | Interobserver  reliability | Test-retest  reliability |
| --- | --- | --- | --- |
| Lateral translation | 0.91 [0.89 to 0.93] | 0.82 [0.68 to 0.91] | 0.88 [0.77 to 0.93] |
| Lateral absolute translation | 0.89 [0.86 to 0.91] | 0.63 [0.39 to 0.79] | 0.75 [0.56 to 0.87] |
| Lateral strain | 0.5 [0.42 to 0.58] | 0.38 [0.07 to 0.63] | 0.36 [0.06 to 0.61] |
| Lateral absolute strain | 0.94 [0.93 to 0.96] | 0.83 [0.69 to 0.91] | 0.82 [0.67 to 0.91] |
| Lateral absolute shear | 0.94 [0.92 to 0.95] | 0.84 [0.7 to 0.91] | 0.85 [0.72 to 0.92] |
| Von Mises strain | 0.93 [0.91 to 0.95] | 0.77 [0.55 to 0.88] | 0.76 [0.58 to 0.87] |

**Table S3. Intraobserver, interobserver and test-retest measured reliability with bootstrapped 95% confidence intervals for elastography parameters**

| Elastography parameters | Intraobserver  reliability | Interobserver  reliability | Test-retest  reliability |
| --- | --- | --- | --- |
| Lateral translation | 0.91 [0.88 to 0.93] | 0.82 [0.69 to 0.9] | 0.88 [0.76 to 0.93] |
| Lateral absolute translation | 0.89 [0.86 to 0.92] | 0.63 [0.38 to 0.8] | 0.75 [0.52 to 0.86] |
| Lateral strain | 0.5 [0.43 to 0.57] | 0.38 [0.17 to 0.56] | 0.36 [-0.08 to 0.63] |
| Lateral absolute strain | 0.94 [0.92 to 0.96] | 0.83 [0.65 to 0.91] | 0.82 [0.63 to 0.89] |
| Lateral absolute shear | 0.94 [0.92 to 0.95] | 0.84 [0.68 to 0.91] | 0.85 [0.72 to 0.9] |
| Von Mises strain | 0.93 [0.91 to 0.95] | 0.77 [0.56 to 0.88] | 0.76 [0.55 to 0.87] |

**Table S4. Mean bias and 95% limits of agreement for interobserver and test-retest measured reliability for elastography parameters**

|  | Interobserver reliability | | Test-retest reliability | |
| --- | --- | --- | --- | --- |
|  | Mean bias and 95% confidence interval | Limits of agreement and 95% confidence interval | Mean bias and 95% confidence interval | Limits of agreement and 95% confidence interval |
| Lateral translation | -0.20  [-0.78 to 0.39] | -4.41  [-5.52 to -3.61]  4.02  [3.22 to 5.13] | -0.49  [-0.98 to 0.01] | -4.06  [-4.99 to -3.38]  3.09  [2.41 to 4.02] |
| Lateral absolute translation | -2.31  [-5.17 to 0.55] | -22.0  [-27.5 to -18.0]  17.4  [13.4 to 22.9] | -0.09  [-2.32 to 2.14] | -16.0  [-20.3 to -12.9]  15.8  [12.8 to 20.1] |
| Lateral strain | -7.49  [-13.14 to -1.85] | -47.7  [-58.4 to -39.9]  32.7  [24.9 to 43.5] | -5.22  [-9.37 to -1.07] | -39.6  [-47.1 to -34.1]  29.2  [23.6 to 36.7] |
| Lateral absolute strain | -10.2  [-30.2 to 9.7] | -141  [-180 to -112]  120  [91 to 160] | 19.3  [1.2 to 37.4] | -105  [-140 to -80]  144  [119 to 179] |
| Lateral absolute shear | -15.7  [-78.2 to 46.8] | -431  [-554 to -343]  400  [311 to 523] | 44.7  [-9.2 to 98.7] | -346  [-448 to -272]  436  [362 to 538] |
| Von Mises strain | -57.8  [-99.7 to -15.9] | -339  [-421 to -280]  223  [164 to 305] | 23.8  [-21.6 to 69.1] | -283  [-371 to -219]  330  [267 to 419] |

**Figure S1. Example schematic diagram of study protocol and interventions in a sample patient.**


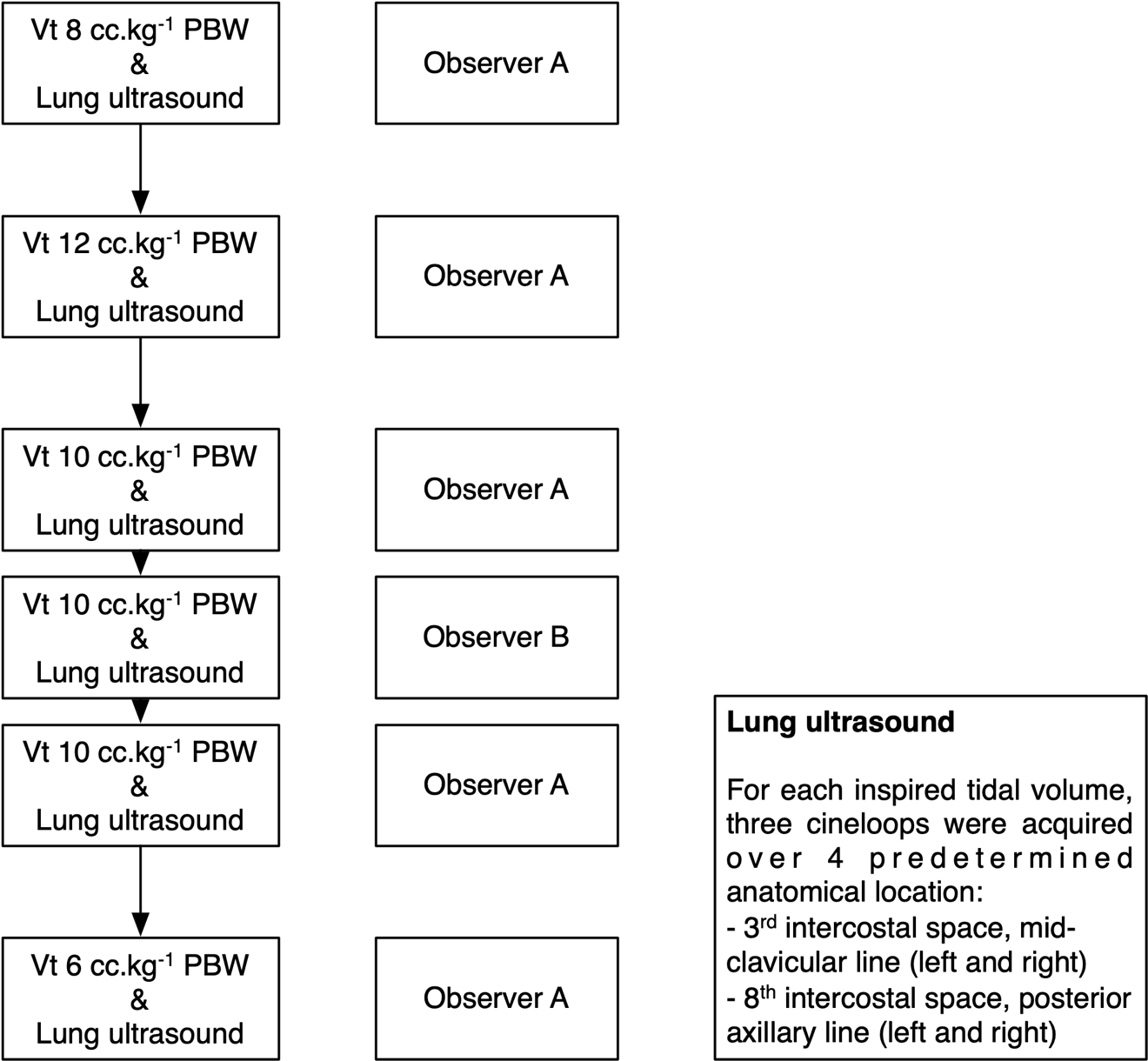


**Figure S2. Pleura segmentation process.** A: All cineloops are reconstructed from radiofrequency data. B: The pleura is segmented manually on a single frame. C: A region of interest of a fixed depth of 2 mm is defined with the segmented pleura forming the upper boundary.


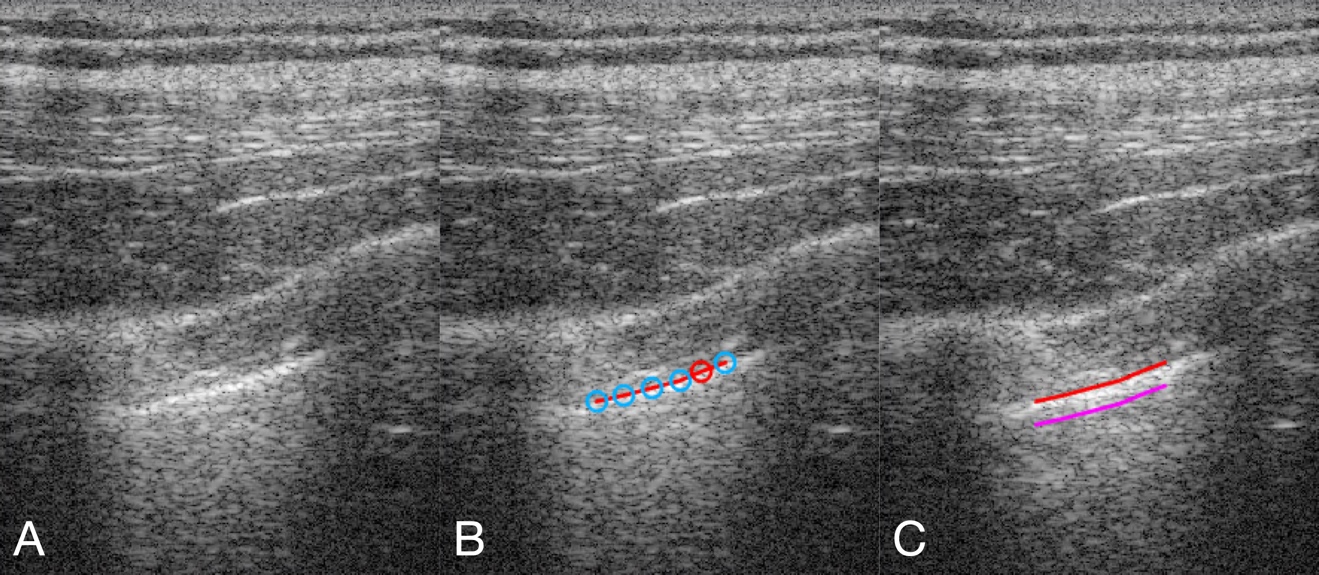


**Figure S3. Schematic representation of tissue axial and lateral translation, strain and shear.**


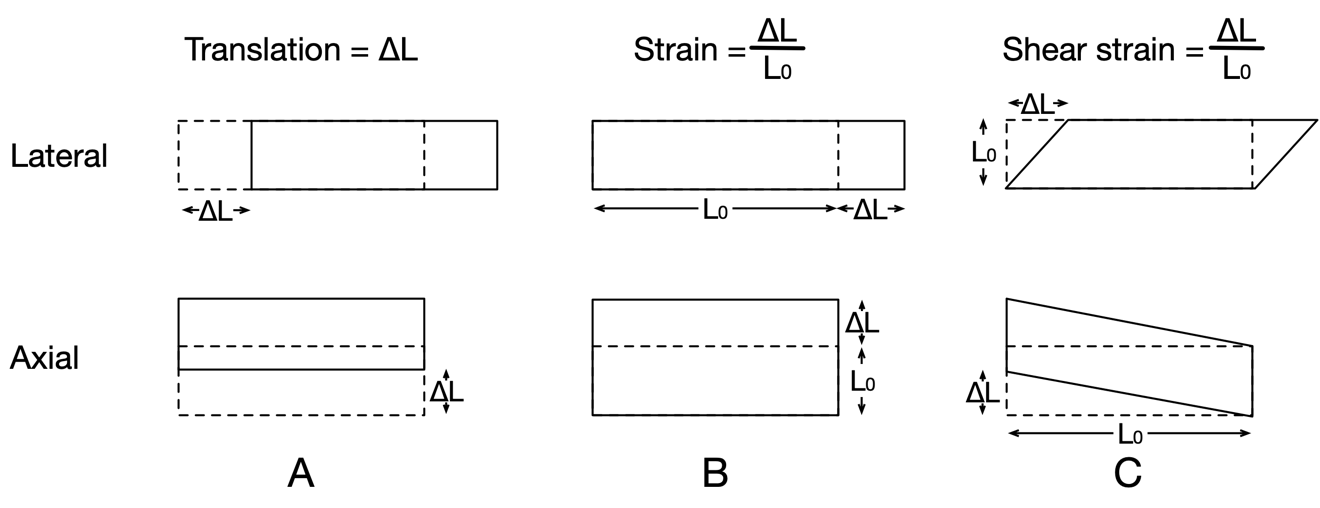


**Figure S4. Study inclusion/exclusion flow diagram.**


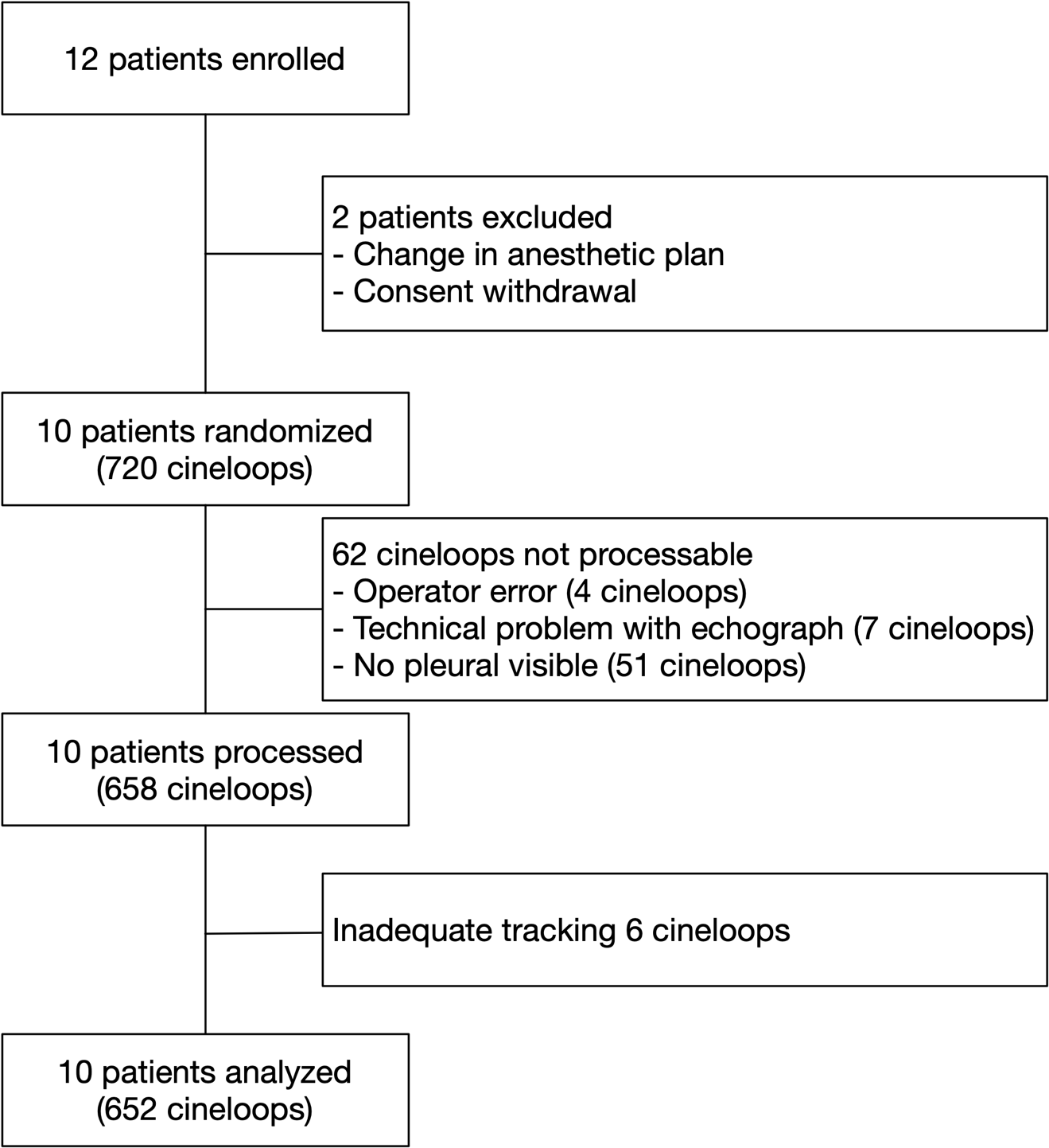


**Figure S5. Slope estimates for elastography parameters** in increasing order stratified by gravity dependence for the first sensitivity analysis. Significant parameters are identified by an asterix.


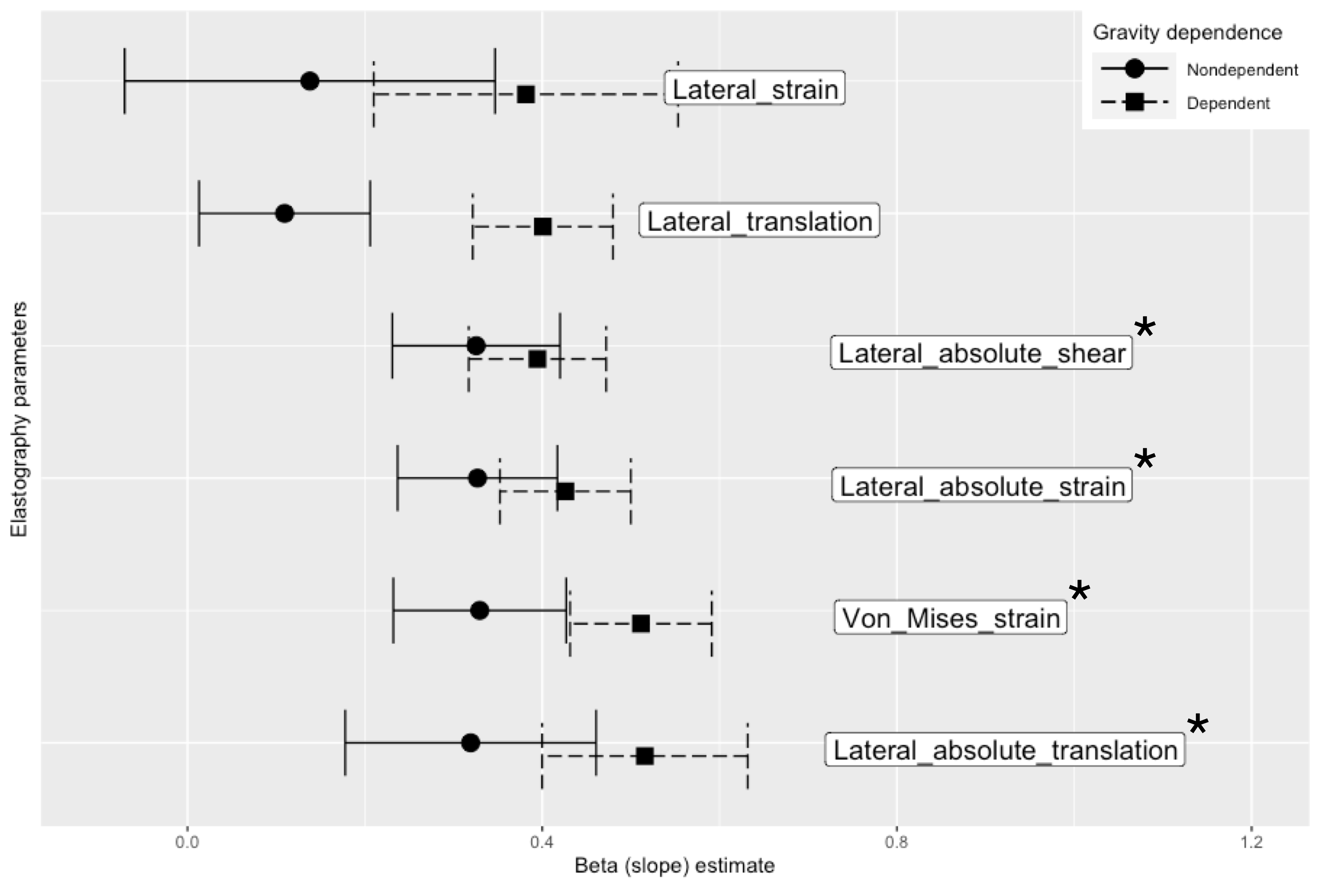


**Figure S6. Bland-Altman plots for intraobserver, interobserver and test-retest reliability measurements for all 6 elastography parameters.** In the left column, Bland-Altman plots for all triplicates measurements (trial 1, 2 and 3) performed by the first observer. Differences between individual measurements and their mean were plotted against their mean. In the center and right columns, Bland-Altman plots for interobserver and test-retest reliability measures. Orange bands represent the 95% confidence intervals of the lower and upper 95% limits of agreement while gray bands represent the 95% confidence interval of the mean bias.


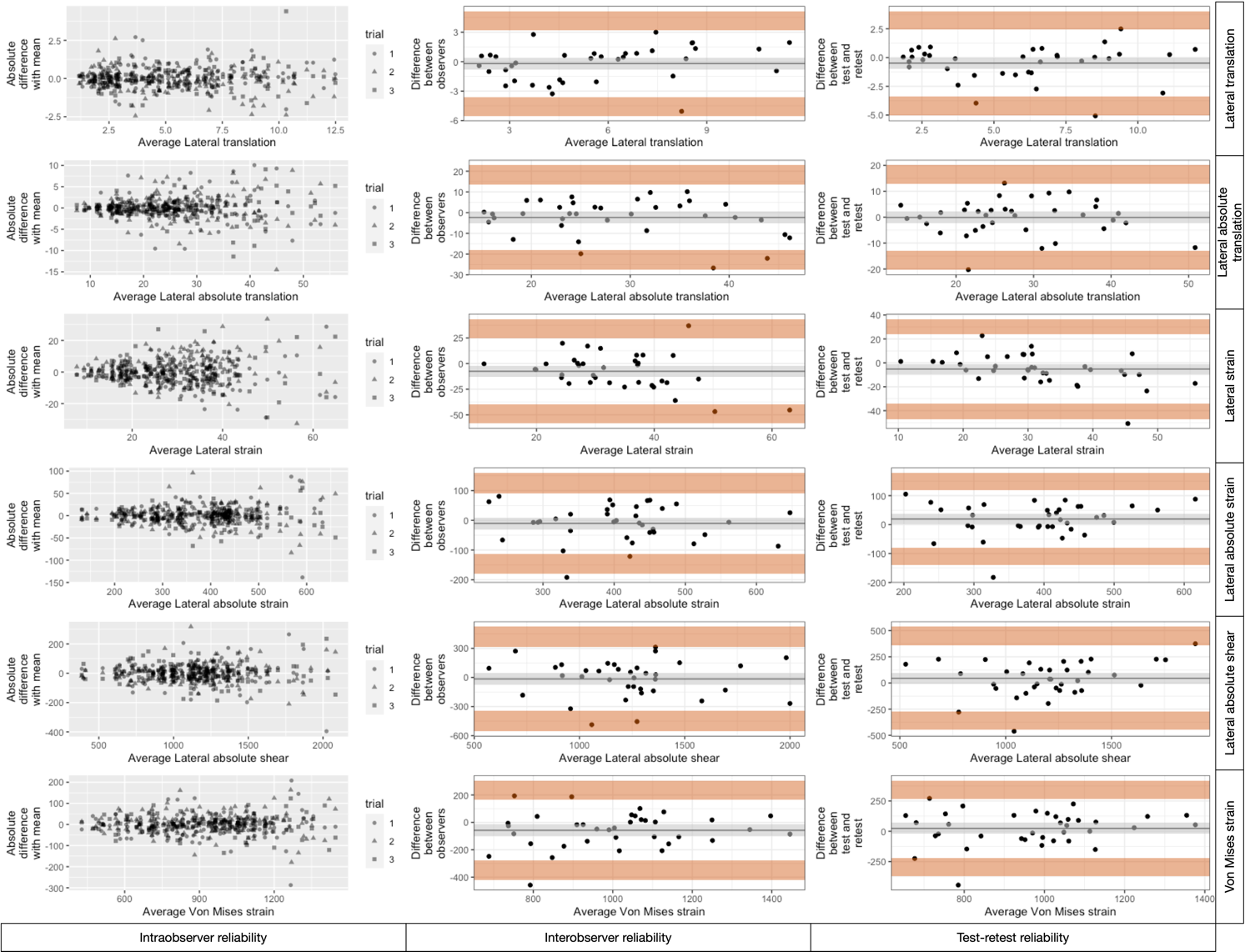


**Figure S7. Reverberation artefacts (white asterix) generated by the visceral pleura** during normal lung sliding lead to the seashore sign when imaged in M-mode. When a pneumothorax occur, loss of reverberation from the separation of the parietal and visceral pleura leads to the barcode sign. The transition between both states (white arrow) is called a lung point.


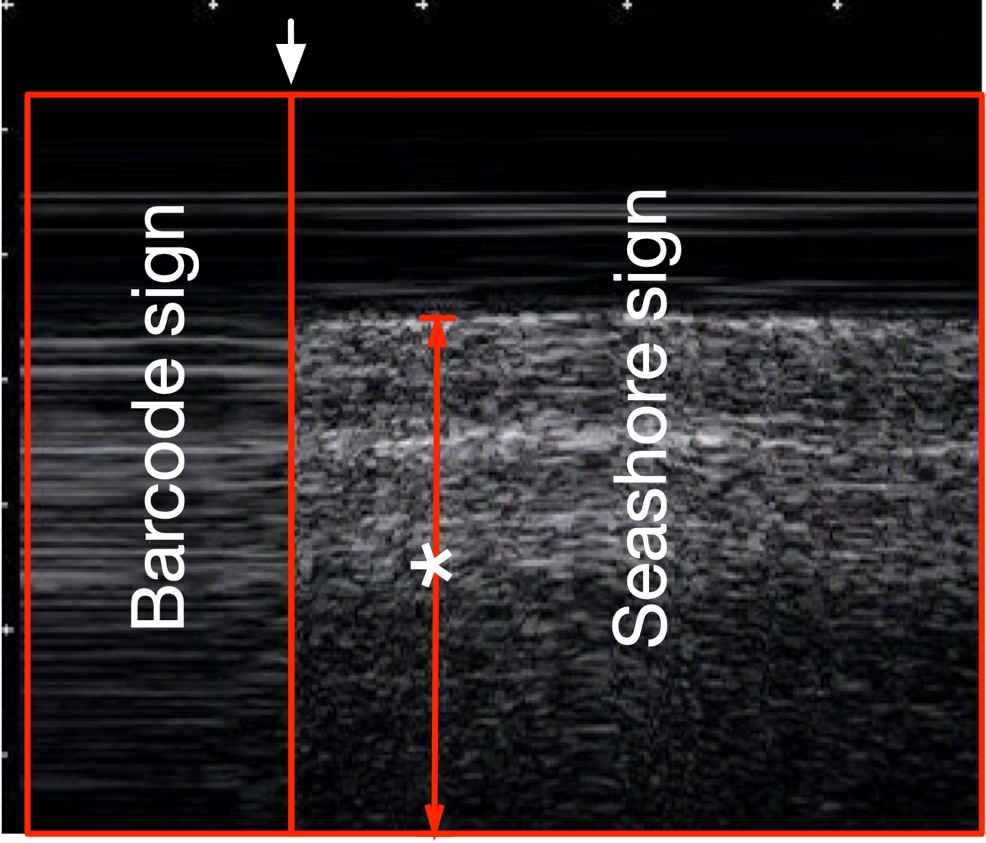


**Figure S8. Example compliance map during lung inflation in a heathy pig under general anesthesia at PEEP of 5 cm H_2_O**. Areas of regional lung inflation are colored in red while areas of regional lung deflation are colored in turquoise. Modified after Perchiazzi G, Rylander C, Derosa S, et al. *Respir Physiol Neurobiol*. 2014;201:60-70


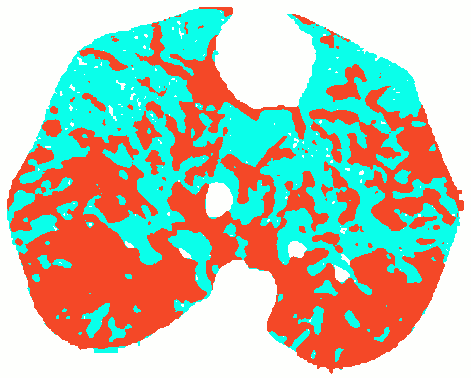

Supplement: Supplementary file 1 [file Data_Sheet_1.DOCX]
